# Evaluation of the Antioxidant Capacities of Antarctic Macroalgae and Their Use for Nanoparticles Production

**DOI:** 10.3390/molecules26041182

**Published:** 2021-02-23

**Authors:** N. González-Ballesteros, M. C. Rodríguez-Argüelles, M. Lastra-Valdor

**Affiliations:** 1 Departamento de Química Inorgánica, CINBIO, Universidade de Vigo, 36310 Vigo, Spain; noeliagb@uvigo.es; 2 Centro de Investigación Marina, Universidade de Vigo, 36331 Vigo, Spain; mlastra@uvigo.es

**Keywords:** *Desmarestia antarctica*, *Iridaea cordata*, green synthesis, gold nanoparticles, silver nanoparticles, reducing power, total phenolic content, DPPH scavenging activity

## Abstract

Macroalgae are sources of bioactive compounds that are interesting from both a chemical and a medical point of view. Although their use in biomedicine has increased significantly in recent years, tests conducted to date have been mostly related to species from temperate latitudes, with the potential application of Antarctic biodiversity being minor. The wide variety of algae species present on Antarctic coastal areas can be a source of new antioxidants. Bearing this in mind, the brown macroalgae *Desmarestia antarctica* (DA) and the red *Iridaea cordata* (IC) were selected for the preparation of aqueous extracts with the aim of analyzing their antioxidant activity. This analysis was performed by determining reducing power, total phenolic content, and 2,2-diphenyl-1-picrylhydrazyl free radical scavenging activity. Furthermore, both extracts were employed to synthesize gold and silver nanoparticles. The nanomaterials were fully characterized by means of UV-Visible spectroscopy, transmission electron microscopy, Z potential measurements, and Fourier transform infrared spectroscopy, which confirmed the formation of stable, spherical nanoparticles with mean diameters of 13.7 ± 3.1 and 17.5 ± 3.7 nm for Ag@DA and Ag@IC and 12.6 ± 1.9 and 12.3 ± 1.6 nm for Au@DA and Au@IC. Antioxidant assays were performed after the synthesis of the nanomaterials to evaluate their possible synergistic effect with the extracts. The results suggest that polysaccharides and proteins may play a key role in the process of reduction and stabilization. Finally, for the sake of comparison, the results obtained for the Antarctic macroalgae *Desmarestia menziesii* and *Palmaria decipiens* have also been considered in the present work.

## 1. Introduction

The Antarctic Ocean is characterized by high levels of biodiversity, both in fauna and flora [1,2]. The progressive isolation of the continent over the last 60 million years seems to account for its highly frequent biotic endemism and species richness [3,4]. Vast subtidal habitats of the rocky substrates of the Western Antarctic Peninsula are occupied by dense and diverse macroalgae assemblages that support productivity and ecosystem functioning [5].

Macroalgae are sources of bioactive compounds that are interesting from both a chemical and a medical point of view due to their relevant effects on human health [6,7]. Although the body of knowledge about algal applications in biomedicine has increased significantly in recent years, this approach is mostly related to species from temperate latitudes [8], and the number of studies on the potentiality of the biodiversity of Antarctic species still low [9]. As a result of the unique environment that Antarctic organisms need to face, we hypothesize that Antarctic macroalgae could be a potential source of new compounds with promising application in biomedicine and suitability for synthetizing new materials, including metallic nanoparticles.

Following our research on the synthesis of nanomaterials by green methods, this study evaluates both the potential to synthesize gold and silver nanoparticles and the antioxidant activity of two Antarctic macroalgae, *Desmarestia antarctica* (DA) and *Iridaea cordata* (IC), which belong to two of the most dominant taxonomic groups in the Antarctic and sub-Antarctic ecoregion, the Orders Desmarestiales and Gigartinales, respectively [10]. Both species are widely distributed along the shallow shores and bays of the Antarctic Peninsula [10,11]. *Iridaea. cordata* is an opportunistic, dry-adapted, red macroalgae that is commonly found in Antarctic waters, but which also grows in the upper sublittoral zones of warmer latitudes, such as the south shores of Argentina, Chile, Australia, and New Zealand [12]. As happens in all red algae, the components of the cell wall of IC are agar, carrageenan, xylans, lectin, and cellulose [12,13]. Recently, it has been studied as a source of polyunsaturated fatty acids [14] and as a biosorbent that can effectively remove crystal violet and methylene blue dyes from aqueous solutions [12]. Lastly, some studies have shown the potential antimicrobial and antitumoral activity of IC extracts and IC polysaccharides [7,15].

*D. antarctica* is a brown macroalgae harvested in the Antarctic Peninsula. *Desmarestia* species are relatively common in the polar and coldtemperate regions of the southern and northern hemispheres. Unfortunately, studies dealing with the composition and application of this species are scarce [16,17,18].

Among other noble metal nanoparticles, gold and silver have attracted tremendous attention over the last few decades because of their exciting physico-chemical properties. Gold nanoparticles can be an excellent starting point for novel biological and chemical applications due to their easy synthesis and functionalization, good biocompatibility, high surface-to-volume ratio, high extinction coefficients, and powerful distance-dependent optical features [19,20]. Silver nanoparticles present other distinctive characteristics, such as high electrical and thermal conductivity, chemical stability, catalytic activity, size and shape dependence, and non-linear optical behavior [21,22]. However, the most remarkable characteristic of silver nanomaterials is their strong antimicrobial activity [23].

There is a recent trend in nanotechnology that consists of the evaluation of a possible synergic effect between the nanomaterials selected and natural biomolecules. Among them, natural antioxidants have attracted considerable attention since they can act against oxidative stress, which has been shown to be an important factor in the appearance and evolution of many diseases, such as diabetes, cardiovascular diseases, cancer, Parkinson’s and Alzheimer’s diseases, arthritis, and even aging [24]. 

In this study, the in vitro antioxidant activity of DA and IC extracts was determined and compared with the results obtained in previous studies conducted for two others Antarctic macroalgae, *Desmarestia menziesii* (DM) and *Palmaria decipiens* (PD) [9]. Then, the antioxidant activity of the four seaweed extracts after the synthesis of gold and silver nanoparticles was analyzed.

## 2. Results and Discussion 

### 2.1. Synthesis and Characterization of Gold and Silver Nanoparticles

Several reaction conditions were tested for the process of synthesis of the nanoparticles by modifying the extract concentrations, the metal salt concentration, temperature, and time of the reaction. The optimal conditions were established after the study of the UV-Vis spectra obtained for the different reactions performed. In all cases, during the synthesis, a change in color was perceived after the reduction of the metal salt. The color changed to red/purple in the case of gold nanoparticles and to yellow/orange in the case of silver nanoparticles.

**Figure 1:**
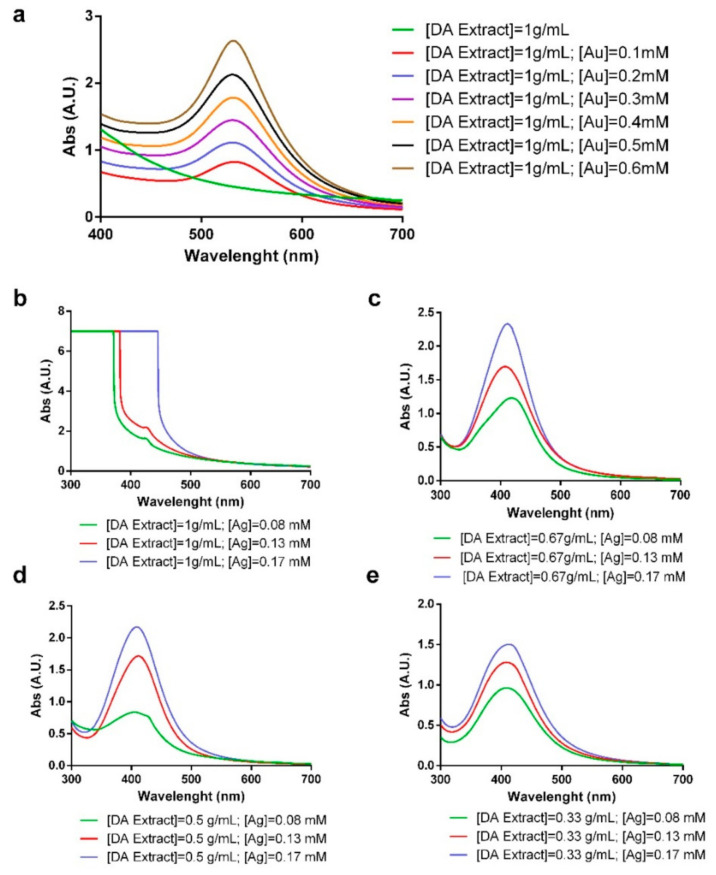
(**a**) UV-Visible spectrum of AuNP synthesized with a fixed concentration of *Desmarestia antarctica* (DA) and different concentrations of HAuCl_4._ (**b**–**e**) UV-Visible spectra of the synthesis of AgNP with different concentrations of DA extract and AgNO_3_. The final optimal reaction conditions are shown in Table 1, while Figure 2a,b show the corresponding UV-Vis spectra.

Figure 1a shows the UV-Vis spectra of gold nanoparticles for a fixed concentration of DA extract and different concentrations of HAuCl_4_. In all the cases, the appearance of the surface plasmon resonance (SPR) band of gold at around 500 nm can be observed, while in the spectra of the extract, there is no band. It can be observed how the SPR band varies depending on the gold concentration. It can be noted that with the lowest concentration of gold tested, the bands that appeared were broad and not really intense. When a higher concentration was added, the band became narrower and more intense. In addition, a variation in the λ_max_ of the SPR band was observed, which shifted to lower values when the concentration of gold was increased. With the highest concentrations of gold, the particles obtained were not stable and tended to aggregate and precipitate.

Regarding silver nanoparticles, Figure 1b–e show the UV-Vis spectra of their synthesis led by DA extract. The reactions were performed at 100 °C in order to synthesize more homogeneous nanoparticles in a faster reaction, since it was observed that at lower temperatures, the reactions did not take place or heterogeneous nanoparticles were obtained. In the UV-Vis spectra, we observed the difference on the characteristic SPR band of silver at around 400 nm when the extract/water ratio is changed and the concentration of silver (Ag) is modified. Figure 1b shows that the characteristic SRP band of silver nanoparticles does not appear when employing the concentrated extract with any of the Ag tested. When diluting the extract, the SPR band appeared for all the silver concentrations.

**Figure 2:**
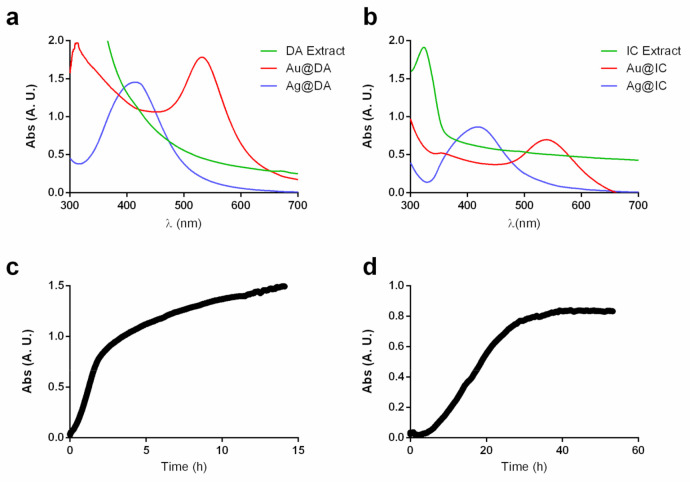
UV-Vis spectra of (**a**) DA extract, Au@DA and Ag@DA and (**b**) *Iridaea cordata* (IC) extract, Au@IC and Ag@IC at the optimal reaction conditions. Time course spectra measurements of (**c**) Au@DA and (**d**) Au@IC at the maximum wavelength of the SPR band.

The spectrum of the synthesized Ag@DA displayed a characteristic SPR band with λ_max_ at 417 nm, while Au@DA showed an intense SPR band at 532 nm, as it can be observed in Figure 2a.

With the red seaweed *I. cordata*, in order to attain a narrow size distribution, it was necessary to further decrease the concentration of the extract, which, as previously mentioned, already had a lower concentration than that of the other macroalgae studied. After several trials, a narrow SPR band with a maximum wavelength at 539 nm was obtained for Au@IC, while Ag@IC showed the characteristic SPR band with λ_max_ at 418 nm, as shown in Figure 2b.

To accurately determine when the gold nanoparticles synthesis was completed, measurements of the absorbance in the maximum wavelength were acquired. Figure 2c shows the spectrum obtained for Au@IC, and it can be noted that the reaction started when HAuCl_4_ was added. A quick increase in absorbance, which corresponds to the change in color, was observed between 0 and 5 h. After that, the reaction slowed down, and a slight increase in absorbance was observed without color change progression. The measurements were stopped after 15 h.

In the case of Au@IC (Figure 2d), it can be observed that the use of a lower concentration of the extract could have affected negatively the kinetics of the reaction. The reaction might be divided into three stages. The first one, between 0 and 5 h, corresponds to the activation process. Next, an increase in absorbance appeared between 5 and 25 h, which corresponds to the change in color observed and the time when the nucleation process might take place. After that, a stabilizing process occurred. The reaction was stopped after 60 h.

pH was measured at room temperature prior to and after the synthesis of nanoparticles, as shown in Table 2. It must be noted that a decrease in pH was observed in all cases, although it is worth mentioning that during the synthesis of gold nanoparticles, the decrease in pH was more significant than in the case of silver nanoparticles. These results are in accordance with previous reports that suggest the involvement of hydroxyl or amino in the reduction of gold(III) and silver(I) to gold(0) and silver(0). The reaction proposed involved the oxidation of hydroxyl or amino groups, which will lead to the liberation of protons and therefore the decrease of pH [6,9].

The Z potential values obtained for the samples are collected in Table 2, where it can be seen that the particles carry a negative electrostatic surface charge. This result suggests the involvement of the anionic polysaccharides present in the extract in the stabilization process of the nanoparticles, since the negative values obtained are in accordance with other studies where marine polysaccharides were employed for the synthesis of nanoparticles [25,26]. It is interesting to highlight the values obtained for the nanoparticles synthesized since, according to Z potential guidelines, nanoparticle dispersions with Z potential values ˃±30 mV are highly stable [27]. When samples are preserved at 4 °C, their stability in the long term (>6 months) has proven to be high.

**Figure 3:**
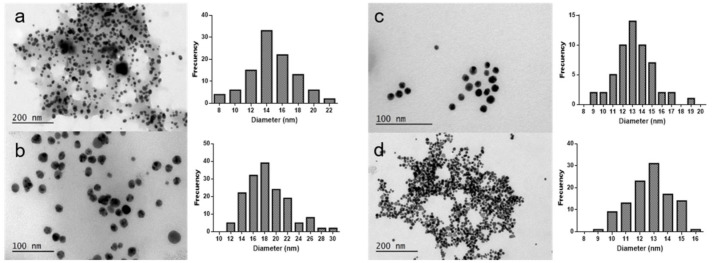
TEM images of (**a**) Ag@DA (**b**) Ag@IC (**c**) Au@DA, and (**d**) Au@IC.

TEM images obtained for the characterization of the silver and gold nanoparticles synthesized by the Antarctic macroalgae are shown in Figure 3. Regarding silver nanoparticles, all the samples analyzed showed the presence of spherical nanoparticles, as seen in Figure 3a,b. Ag@DA had a mean diameter of 13.7 ± 3.1 nm (Figure 3a). The nanoparticles obtained with DM using the same reaction conditions were bigger, with a mean size of 17.8 ± 2.6 nm [9]. The mean diameter of silver nanoparticles obtained with the red algae *Iridaea cordata* (Figure 3b) is similar to that obtained with the brown algae DM, with sizes of 17.5 ± 3.7 nm. The smallest nanoparticles were obtained with PD extract, being the only silver nanoparticles synthesized in the context of the present study with a diameter lower than 10 nm [9]. Previously, the stability of these nanoparticles had been confirmed with the determination of the Z potential (Table 3). This stability can be due to the fact that the nanoparticles are embedded in the extract matrix, as Figure 3a,b clearly illustrate, even after the centrifugation and purification step in the TEM sample preparation.

In the case of the gold nanoparticles synthesized, the extract cannot be clearly observed in the images acquired (Figure 3c,d). In the case of Au@IC, this could be due to the lower concentration of extract employed for the synthesis and the centrifugation step for TEM sample preparation. A predominance of spherical nanoparticles was observed in both samples, and the mean diameters obtained were 12.6 ± 1.9 and 12.3 ± 1.6 nm for Au@DA and Au@IC, respectively. These sizes are very similar to those obtained with the brown algae *D. menziesii*, with mean diameters of 11.5 ± 3.3 nm, while in the case of Au@PD, the nanoparticles obtained were the biggest ones, with mean diameters of 36.8 ± 5.3 nm [9].

**Figure 4:**
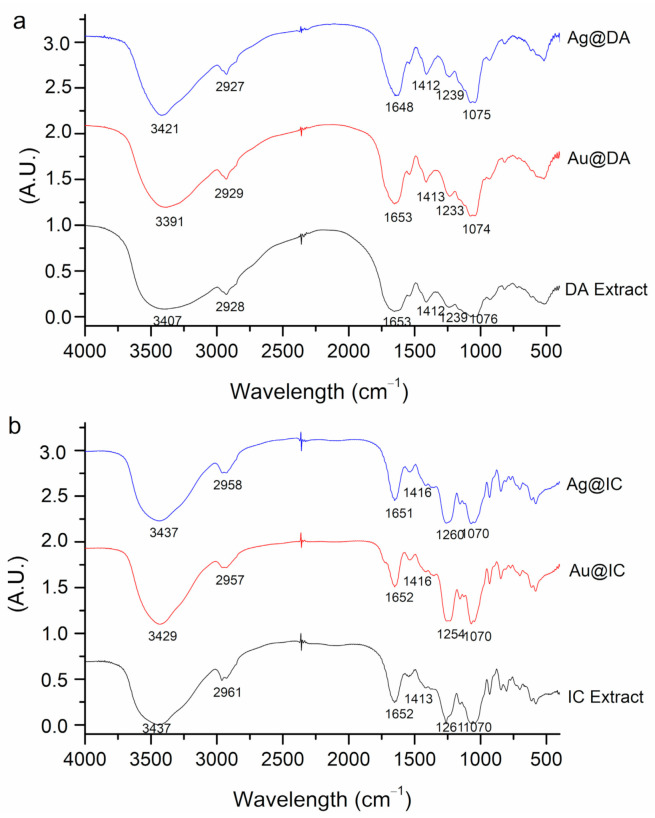
Fourier transform infrared spectra of (**a**) DA extract and (**b**) IC extract before and after nanoparticles were synthesized.

In this study, the FTIR technique was employed for the evaluation of the changes observed in the functional groups of the molecules in the extracts before and after the nanoparticles were synthesized. Figure 4a,b show the spectra and assignation of bands for DA and IC samples, respectively. For the assignation of the bands, previous studies were used as reference, mostly from the same species, but also from the same genus or other Antarctic species [9,12,28,29,30].

In general, O-H stretching vibrations of the hydroxyl group in alcohols and N-H stretching vibrations in amides and amines are assigned to the broad band that appeared in all spectra between 3437 and 3391 cm^−1^, while the weaker signal at 2961–2927 cm^−1^ could be related to C-H stretching vibrations of the hydrocarbon chains. Carboxylate groups are typically assigned to two bands; one more intense at around 1653–1648 cm^−1^, corresponding to an asymmetrical stretching, and a weaker band at 1412–1416 cm^−1^, which is assigned to symmetrical stretching from amide I and II of proteins. The sugar ring and glycosidic bond C-O stretching vibrations might account for the signals at 1070 cm^−1^. The C-O-S bending vibration observed at 800 cm^−1^ and S-O stretching vibration at 1260 cm^−1^ ascribed to sulfated esters point to the presence of sulfate groups in the polysaccharide structure. Some studies on brown seaweed suggest that the band at 800 cm^−1^ is characteristic of mannuronic acid residues [28,29].

It must be noted that in the case of IC extract, the band at 1200 cm^−1^ is more intense than in the case of DA extract. According to some studies, the intensity of this band is an indicator of the degree of sulfurization of the polysaccharides [31].

When comparing DA extract with Au@DA and Ag@DA, a different behavior related to the shifts in bands could be observed. Firstly, in the case of Au@DA, the peak of 3400 cm^−1^ shifted to lower wavelengths, while in Ag@DA, it shifted to higher wavelengths. These shifts might indicate an involvement of either the hydroxyl functional groups from polyphenols and polysaccharides or the amino groups of proteins in the bioreduction of the salts employed. This could be related to other studies that have synthesized nanoparticles using alginate, which is a polysaccharide isolated from brown seaweed [32].

The peak at 1600 cm^−1^, which corresponds to carbonyl stretching, also shifted to lower wavelengths in Ag@DA, but there was no change in Au@DA. This is in consonance with other studies, which have suggested that the carbonyl group from proteins can effectively bind metals, so it is likely that proteins could cap silver nanoparticles to impede agglomeration [33,34].

Lastly, significant differences were observed in both Ag@DA and Au@DA in the bands between 1200 and 1000 cm^−1^ when compared with DA extract. The shifts to lower wavelengths and differences in intensity observed could indicate a role of the sulfonic groups from polysaccharides in metal binding.

Regarding IC extract, when compared with Au@IC and Ag@IC, there were minor shifts in the position, shape, and intensity of the bands at 3429 cm^−1^ and in the region between 1400 and 1200 cm^−1^ in the case of Au@IC. On the other hand, no major changes were observed in Ag@IC, with just some differences in the intensity and broadness of the bands.

### 2.2. In Vitro Antioxidant Activity

**Figure 5:**
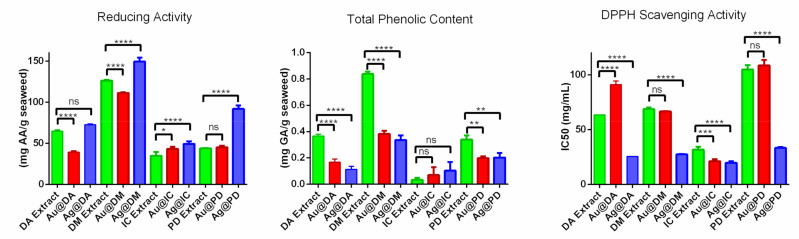
Graph bar showing the results of reducing power, total phenolic content, and 1,1-diphenyl-2-picryl-hydrazyl (DPPH) scavenging activity of DA, DM, IC and PD extracts before and after the synthesis of gold and silver nanoparticles. n.s. *p* > 0.05, * *p* ≤ 0.05, ** *p* ≤ 0.01, *** *p* ≤ 0.001, **** *p* ≤ 0.0001.

In the present study, the antioxidant activity of DA and IC extracts was determined and compared with the values obtained for the two seaweed previously studied, *D. menziesii* and *P. decipiens* [9]. Furthermore, the antioxidant activity of the four macroalgae extracts after the synthesis of nanoparticles was also analyzed. The values of the reducing activity, total phenolic content, and radical scavenging activity of the aqueous extracts of *D. antarctica*, *D. menziesii, I. cordata,* and *P. decipiens* are represented in Figure 5.

It can be observed that DM possesses the highest reducing power, with a value that doubles that of the other *Desmarestia.* When compared with the red seaweed, DM presents a reducing power that triples that of *P. decipiens* and is four times that of *I. cordata.* The reducing power of the two red seaweed is lower than that of the brown seaweed, which is in line with other studies where this relation was maintained [35,36].

Regarding the total phenolic content (TPC) of the samples, it can be observed that the direct relationship that is usually noticed between antioxidant activity and TPC is not maintained. It can be noted that DM extract possesses the highest TPC, with 0.84 ± 0.02 mg gallic acid equivalents (GAE)/g alga. However, *D. antarctica* and PD, which show quite different values of reducing power, do not show significant differences as regards phenolic content, with values of 0.37 ± 0.01 and 0.34 ± 0.03 mg GAE/g alga, respectively. Surprisingly, the value of TPC obtained for *I. cordata* is extremely low, to the extent of being negligible.

In the case of 1,1-diphenyl-2-picryl-hydrazyl (DPPH) free radical scavenging, the lowest IC_50_ value was obtained with *I. cordata*, which, in contrast, has the lowest TPC value of the seaweed studied. Both *Desmarestia* show similar IC_50_ values, 63.3 ± 0.2 mg/mL for DA and 68.7 ± 1.3 mg/mL for DM. PD has the highest IC_50_ value, almost double those of both *Desmarestia,* indicating a lower scavenging activity.

In the literature, information about these species of seaweed is scarce. The studies found focus on the content of lipids and fatty acid composition, as well as on polysaccharides [37], and they provide data related to the content of hydrocarbons in these species [38,39,40,41]. For instance, the study conducted by Dhargalkar and Bhosle shows that *D. menziessi* has a higher content of hydrocarbons than *P. decipiens* but no significant difference in lipids [40]. The higher content of polysaccharides would account for the more significant reducing power of *D. menziesii* in comparison with *P. decipiens*. *D. menziesii* also shows higher reducing power than *D. antarctica*, which could be related to the higher content of phlorotannins described by Iken et al. [42]. 

After the synthesis of the nanoparticles, the reducing power, TPC, and DPPH scavenging activity of the extracts were also determined. In Figure 5, the results obtained for DA extract, Au@DA, and Ag@DA are shown. As regards reducing power, gold and silver nanoparticles behave differently. Au@DA possesses almost half the reducing power after the synthesis, while the value obtained for Ag@DA does not show significant differences compared to that of the extract before the synthesis. Interestingly, in both cases, a significant decrease in the TPC can be observed, which suggests an active role of the phenolic compounds present in DA extract in the reduction process for the synthesis of the nanoparticles. In the case of the DPPH, the same behavior as in the case of the reducing power is observed. The IC_50_ value of Au@DA is higher after the synthesis, while it is lower in the case of Ag@DA. 

When the values obtained for DM extract are compared to those obtained after the synthesis of Au@DM and Ag@DM, the results obtained showed a similar pattern to that of the results obtained with the other *Desmarestia*. A decrease in the reducing power was observed in Au@DM, while a significant increase was observed in Ag@DM. Regarding TPC, it is noteworthy that it decreased by half in both samples. The IC_50_ values calculated for the DPPH scavenging activity showed a significant decrease for Ag@DM, while there was no significant difference for Au@DM. 

Regarding the comparison of IC extract before and after the synthesis, the results obtained showed a slight increase in the reducing power as well as a diminution in the IC_50_ value for both Au@IC and Ag@IC. In both cases, the difference in the total phenolic content was insignificant. Due to the low TPC obtained for IC, it could be argued that that they do not intervene in the synthesis of nanoparticles.

Finally, the results obtained for PD extract did not reveal any significant variations in the reducing power and scavenging activity of Au@PD. On the other hand, a notable increase in the reducing power was observed, and consequently, a decrease in the IC_50_ value was also obtained for Ag@PD. In both cases, there was a significant lowering in the TPC obtained, indicating their contribution during the synthesis of the nanoparticles. 

## 3. Materials and Methods

### 3.1. Preparation of Aqueous Algae Extracts

Samples of the macroalgae *D. antarctica* and *I. cordata* were collected at low spring tide during the austral autumn (February 2017) in Foster Bay in the North Antarctic Peninsula (62°58′53″ S, 60°39′10″ W). The samples were placed in plastic bags and frozen at −20 °C until they were brought to the laboratory. The protocol followed for the preparation of the aqueous extracts was the same as the one used with the Antarctic macroalgae *D. menziesii* and *P. decipiens*, for the sake of comparison [6,9]. In the case of *I. cordata*, it was necessary to modify the ratio of the biomass/water extraction due to the formation of a gel. The final concentration used was 0.2 g/mL.

### 3.2. Synthesis of Gold and Silver Nanoparticles

For the preparation of the nanomaterials, the protocol followed was the one previously reported [9]. The reaction conditions established initially were the same as those used in the case of *D. menziesii* and *P. decipiens*. However, in an attempt to obtain nanomaterials with homogeneous size and shape, the reaction conditions were modified. Test were done changing the concentration of the extracts and maintaining the concentration of metal salt, and also maintaining the concentration of extract and changing the concentration of metal salt. The reaction outcome was monitored by analyzing changes in color and by means of UV-Visible spectroscopy. Table 3 shows the reaction conditions for both silver and gold.

### 3.3. Characterization of Gold and Silver Nanoparticles

First, UV-Vis spectra measurements in a range between 200 and 700 nm of wavelength were acquired to confirm the formation of nanoparticles using a spectrometer Jasco V-670. Once the reaction conditions were determined, the size and shape of nanoparticles were analyzed by transmission electron microscopy, using the model JEOL JEM 1010 (100 kw). Images were acquired with a CCD Orius camera, and data were analyzed employing Digital Micrograph (Gatan) and Image J softwares. For the preparation of the samples, first, they were centrifuged at 10,000 rpm for 30 min; then, the pellet was redispersed in milli Q water using an ultrasound bath for 15 min. After that, the samples were placed in 400 mesh copper grids coated with formvar and carbon. Afterwards, infrared spectroscopical analysis was performed in order to study the composition of the extracts and the changes observed after the synthesis of the nanomaterials. The samples were dried at 80 °C, and KBr pellets were prepared. IR spectra were recorded between 400 and 4000 cm^−1^ in a Jasco FT/IR-6100. The study of the stability of the samples was conducted by Z potential measurements in a Zetasizer Nano S.

### 3.4. Evaluation of the Antioxidant Activity

Three assays were performed to analyze the antioxidant and antiradical activity of DA and IC extracts before and after the synthesis of the nanomaterials. The DPPH radical scavenging activity, the reducing power, and the total content of phenols were determined by the methods previously reported [6,9]. 

Briefly, the DPPH free radical scavenging activity was tested using the protocol previously described [43]. In summary, a 0.1 mM solution of DPPH in methanol was freshly prepared, and 1 mL of this solution was added to 3 mL of the stem extract diluted at a ratio of 1:12. The mixture was thoroughly mixed and allowed to stand at room temperature for 30 min. For the blank preparation, 3 mL milliQ water was used instead of the sample, and a sample control was also made by mixing 3 mL of sample with 1 mL of methanol. Then, the absorbance was measured at 517 nm using a UV-Vis spectrophotometer. Lower absorbance values of reaction mixture indicate higher free radical scavenging activity. The capability of scavenging the DPPH radical was calculated by using the formula:

$$DPPH\;scavenging\;effect\;\left(\%\;inhibition\right)=\left(1-\frac{A_{s}-A_{s0}}{A_{b}}\right)\times 100$$

where *A_b_* is the absorbance of the blank, *A_s_* is the absorbance of the extract samples, and *A_s_*_0_ is the absorbance of the control. All the tests were performed by triplicate, and the results were averaged. The results are expressed as the concentration required to inhibit the radical concentration of DPPH by half (IC_50_).

TPC was analyzed by means of a modified version of the Folin–Ciocalteau method [6]. First, 100 µL of sample were mixed with 2 mL of sodium carbonate 5% and left to stand for 2 min. Then, 100 µL of Folin–Ciocalteau reagent 50% were added and thoroughly mixed. The mixture was allowed to stand for 30 min at room temperature in the dark. The reduction of the reagent by phenolic compounds formed a blue compound, and the absorbance was measured at 725 nm. Results are expressed as gallic acid equivalents (GAE) per gram of stem, using a calibration curve over the range of 0.05–1 mg/mL. All measurements were performed by triplicate, and results are expressed as mean ± standard deviation.

Finally, the reducing power was determined using the method previously reported [6]. First, 1.0 mL of sample was mixed with 2.5 mL of phosphate buffer (0.2 M, pH 6.6) and 2.5 mL potassium ferricyanide (1%). The reaction mixture was incubated for 20 min at 50 °C. Then, 2.5 mL of trichloacetic acid (10%) were added, and the mixture was centrifuged for 10 min at 4000 rpm. Finally, 2.5 mL of the upper layer were mixed with 2.5 mL of milliQ water and 0.5 mL of ferric chloride (0.1%). Then, the absorbance was measured at 700 nm. Higher absorbance indicates a higher reducing activity. A standard curve was built with ascorbic acid at concentrations between 50 and 400 mg/L, and results are expressed as ascorbic acid equivalents (AAE) per gram of stem. All assays were performed by triplicate and presented as mean ± standard deviation.

### 3.5. Statistical Analysis

GraphPad Prism 6 software was employed for the determination of significant differences between the antioxidant activity obtained for the different extracts before and after the synthesis of nanoparticles by performing of a one-way analysis of variance (either an ANOVA or a Kruskal–Wallis test) and a Tukey’s or Dunn’s test afterwards. All experiments were done three times. In the graphs, results are expressed as: n.s. *p* > 0.05, * *p* ≤ 0.05, ** *p* ≤ 0.01, *** *p* ≤ 0.001, **** *p* ≤ 0.0001.

## 4. Conclusions

For the first time, the Antarctic macroalgae *D. antarctica* and *I. cordata* were employed to synthesize gold and silver nanoparticles, using an eco-friendly, cost-effective, one-pot approach. The aqueous extracts of both seaweed were employed for the effective reduction of Ag(I) and Au(III) and the stabilization of the prepared nanoparticles.

The characterization of these nanomaterials by transmission electron microscopy shows a predominance of spherical nanoparticles and that the bigger nanoparticles obtained were Ag@IC with a mean diameter of 17.5 ± 3.7 nm, which was followed by Ag@DA with a mean size of 13.7 ± 3.1 nm. Both gold nanoparticles show a similar size distribution of 12.6 ± 1.9 and 12.3 ± 1.6 nm for Au@DA and Au@IC, respectively. The Z potential values obtained for the four samples show that the nanoparticles carry a negative electrostatic surface charge and that they are highly stable. FTIR spectra analysis shows the functional groups of components of the extracts before and after the synthesis of the nanoparticles. The results suggest that polysaccharides and proteins may play a role in the process of reduction and stabilization. 

Finally, the in vitro antioxidant activity of the extracts prior to and after the formation of the nanoparticles was analyzed. It was observed that *D. antarctica* presents higher reducing power and total phenolic content than *I. cordata*, while it has lower DPPH scavenging activity. 

## Figures and Tables

**Table 1:** Optimal reaction conditions for gold and silver nanoparticles synthesis.

Algae	[Extract] (g/mL)	[Ag] (mM)	[Au] (mM)	T (°C)	T (h)	SPR λ_max_ (nm)	Code
*D. antarctica*	0.25	0.17	-	100	1	417	Ag@DA
	1	-	0.4	RT	15	532	Au@DA
*I. cordata*	0.2	0.17	-	100	1	418	Ag@IC
	0.13	-	0.5	RT	40	539	Au@IC

**Table 2:** pH and Z potential measurements of the extracts and gold and silver nanoparticles synthesized.

Sample	pH	T (°C)	Z Potential (mV)
***D. antarctica***	6.17	25	-
**Au@DA**	5.05	25	−42.4 ± 2.4
**Ag@DA**	5.96	25	−50.7 ± 2.3
***I. cordata***	7.30	25	-
**Au@IC**	3.87	25	−58.9 ± 1.5
**Ag@IC**	6.17	25	−45.6 ± 1.1

**Table 3:** Reaction conditions employed for the synthesis of gold and silver nanoparticles.

Extract/Water Ratio	[Ag] (mM)	[Au] (mM)
1:0	0.08–0.17	0.1–0.5
0.75:0.25		
0.5:0.5		
0.25:0.75		

## Data Availability

Data is contained within the article.
